# Staurosporine Induces Filamentation in the Human Fungal Pathogen *Candida albicans* via Signaling through Cyr1 and Protein Kinase A

**DOI:** 10.1128/mSphere.00056-17

**Published:** 2017-03-01

**Authors:** Jinglin L. Xie, Teresa R. O’Meara, Elizabeth J. Polvi, Nicole Robbins, Leah E. Cowen

**Affiliations:** Department of Molecular Genetics, University of Toronto, Toronto, Ontario, Canada; Carnegie Mellon University

**Keywords:** *Candida albicans*, cyclic AMP, kinase inhibitor, morphogenesis, staurosporine, virulence

## Abstract

Protein kinases are key regulators of signal transduction pathways that participate in diverse cellular processes. In fungal pathogens, kinases regulate signaling pathways that govern drug resistance, stress adaptation, and pathogenesis. The impact of kinases on the fungal regulatory circuitry has recently garnered considerable attention in the opportunistic fungal pathogen *Candida albicans*, which is a leading cause of human morbidity and mortality. Complex regulatory circuitry governs the *C. albicans* morphogenetic transition between yeast and filamentous growth, which is a key virulence trait. Here, we report that staurosporine, a promiscuous kinase inhibitor that abrogates fungal drug resistance, also influences *C. albicans* morphogenesis by inducing filamentation in the absence of any other inducing cue. We further establish that staurosporine exerts its effect via the adenylyl cyclase Cyr1 and the cyclic AMP (cAMP)-dependent protein kinase A (PKA). Strikingly, filamentation induced by staurosporine does not require the known upstream regulators of Cyr1, Ras1 or Pkc1, or effectors downstream of PKA, including Efg1. We further demonstrate that Cyr1 is capable of activating PKA to enable filamentation in response to staurosporine through a mechanism that does not require degradation of the transcriptional repressor Nrg1. We establish that staurosporine-induced filamentation is accompanied by a defect in septin ring formation, implicating cell cycle kinases as potential staurosporine targets underpinning this cellular response. Thus, we establish staurosporine as a chemical probe to elucidate the architecture of cellular signaling governing fungal morphogenesis and highlight the existence of novel circuitry through which the Cyr1 and PKA govern a key virulence trait.

**IMPORTANCE** The impact of fungal pathogens on human health is devastating. One of the most pervasive fungal pathogens is *Candida albicans*, which kills ~40% of people suffering from bloodstream infections. Treatment of these infections is extremely difficult, as fungi are closely related to humans, and there are limited drugs that kill the fungus without host toxicity. The capacity of *C. albicans* to transition between yeast and filamentous forms is a key virulence trait. Thus, understanding the genetic pathways that regulate morphogenesis could provide novel therapeutic targets to treat *C. albicans* infections. Here, we establish the small molecule staurosporine as an inducer of filamentous growth. We unveil distinct regulatory circuitry required for staurosporine-induced filamentation that appears to be unique to this filament-inducing cue. Thus, this work highlights the fact that small molecules, such as staurosporine, can improve our understanding of the pathways required for key virulence programs, which may lead to the development of novel therapeutics.

## Abstract (precis)

The impact of fungal pathogens on human health is devastating. One of the most pervasive fungal pathogens is *Candida albicans*, which kills ~40% of people suffering from bloodstream infections. Treatment of these infections is extremely difficult, as fungi are closely related to humans, and there are limited drugs that kill the fungus without host toxicity. The capacity of *C. albicans* to transition between yeast and filamentous forms is a key virulence trait. Thus, understanding the genetic pathways that regulate morphogenesis could provide novel therapeutic targets to treat *C. albicans* infections. Here, we establish the small molecule staurosporine as an inducer of filamentous growth. We unveil distinct regulatory circuitry required for staurosporine-induced filamentation that appears to be unique to this filament-inducing cue. Thus, this work highlights the fact that small molecules, such as staurosporine, can improve our understanding of the pathways required for key virulence programs, which may lead to the development of novel therapeutics.

## INTRODUCTION

Protein kinases regulate diverse cellular functions in eukaryotes through the highly regulated propagation and amplification of stimuli via signal transduction cascades that modulate biological responses. As a consequence of the profound impact of protein kinases on cellular signaling, perturbation of kinase-mediated signaling pathways is implicated in diverse diseases, including cancer, diabetes, and inflammation (1, 2). Kinases implicated in human disease provide attractive targets for drug development, as do kinases that enable virulence and drug resistance of microbial pathogens. For example, the protein kinase Pkc1 regulates fungal drug resistance and virulence (3), and the cognate natural product inhibitor cercosporamide displays potent antifungal activity against diverse pathogenic fungi (4). Bioactive natural products have provided an unparalleled source of therapeutic agents, particularly for the treatment of infectious disease. They provide a reservoir of specialized chemical scaffolds that have been selected over the course of evolution to mediate interactions between organisms in the environment. Two of the three classes of antifungals used in the clinic to treat systemic fungal infections are naturally derived, the polyenes and echinocandins. Natural products may provide the next frontier for antifungal drug development, as highlighted by the antifungal activity of the complex macrolactone ibomycin (5), and the cyclohexadepsipeptide beauvericin (6–8). Thus, targeting druggable hubs in signal transduction pathways with natural products holds great potential for the treatment of fungal infections.

The paucity of antifungal drugs and the emergence of drug resistance in fungal pathogens demand the development of novel therapeutic strategies. Fungal pathogens pose a growing threat due to the increasing population of individuals with compromised immune systems, with the current death toll due to fungal infections approaching 1.5 million people per year (9). One of the most common causative agents of fungal infection worldwide is *Candida albicans*, with mortality rates that approach 40% even with current treatments (10). *C. albicans* exists in different morphological states, depending on environmental conditions. It typically grows in the yeast form in rich medium at 30°C and switches to a filamentous form in response to diverse cues, including serum, elevated CO_2_, and nutrient starvation (11, 12). The induction of filamentous growth often requires a concomitant increase in temperature from 30°C to 37°C, with a few notable exceptions being induction of filamentation at 30°C in response to cell cycle arrest or inhibition of the molecular chaperone Hsp90 (13, 14). Importantly, the ability of *C. albicans* to undergo such drastic changes in morphology is a key virulence factor (15–17), providing new opportunities for therapeutic intervention. Although the potential utility of targeting virulence factors has only recently been appreciated, benefits include expanding the repertoire of antifungal targets, minimizing effects on the host mycobiome, and reducing selection pressure for the evolution of drug resistance (18, 19). As such, there is great potential to identify novel drug targets by elucidating the complex circuitry controlling fungal virulence traits.

The capacity for morphological transitions is a key virulence trait in *C. albicans* and is regulated by a multitude of cellular signaling cascades, some of which regulate filamentation in response to diverse cues, while others have more specialized functions. The complexity of the genetic circuitry underlying filamentous growth is highlighted by a recent genome-scale analysis in *C. albicans*, which identified 974 morphogenetic regulators (20). One of the most well-studied pathways that is crucial for filamentation in response to diverse cues is the Ras1-Cyr1-protein kinase A (PKA) pathway, also referred to as the cyclic AMP (cAMP) signaling pathway (12, 21). Ras1 is a GTPase that cycles between an inactive GDP-bound state and an active GTP-bound state. Upon activation by the guanine exchange factor Cdc25, Ras1 stimulates the adenylyl cyclase activity of Cyr1 to produce cAMP. The elevated intracellular cAMP levels activate the catalytic subunits of PKA, Tpk1 and Tpk2, which in turn activate a transcriptional program that governs morphogenesis through transcription factors such as Efg1. More recently, the Lrg1-Rho1-Pkc1 cascade was established as another core pathway that regulates morphogenesis in response to diverse filament-inducing cues, in part via the cAMP signaling pathway (22). Filamentation in response to specific cues is also controlled by more specialized pathways that include the Cek1/Cek2-dependent mitogen-activated protein (MAP) kinase cascade, the Rim101-mediated pH pathway, and the Cek1-dependent embedded pathway, as well through cell cycle arrest regulated by Cdc28 (12). Chemical biology approaches complement functional genomics as a strategy to probe cellular circuitry governing morphogenesis and enable the development of novel therapeutic strategies.

There is a limited repertoire of chemical scaffolds that have been linked to modulating morphogenetic transitions. Fascinating examples include the quorum-sensing molecule farnesol, which *C. albicans* produces at high cell densities in order to repress filamentation (23, 24), and the quorum-sensing molecule pyocyanin, which is produced by the bacterial pathogen *Pseudomonas aeruginosa* and also inhibits* C. albicans* filamentation (25). Molecules that induce filamentation include natural product inhibitors of Hsp90, such as geldanamycin or radicicol, and agents that induce cell cycle arrest, such as nocodazole or hydroxyurea. Given the key role for protein kinases in regulating signaling pathways that control morphogenesis, kinase inhibitors may provide an untapped source of chemical probes to modulate this important virulence trait. One prominent protein kinase inhibitor is the natural product staurosporine, which was originally isolated from the bacterium *Saccharothrix aerocolonigenes* (originally referred to as *Streptomyces staurosporeus*) (26). Through biochemical and genetic approaches in the model yeast *Saccharomyces cerevisiae*, the primary target of staurosporine was identified as Pkc1; however, the molecule also exhibits promiscuous inhibitory activity against other kinases (27–29). Staurosporine has antifungal activity against *C. albicans* (26), which may be attributable to the inhibition of Pkc1, which in turn regulates cellular responses to cell wall and cell membrane stresses and is required for *C. albicans* proliferation in the mouse model of systemic infection (3, 30).

In this study, we have established that staurosporine induces robust filamentous growth in *C. albicans* at 30°C and does so independent of inhibition of Pkc1. Filaments formed in response to staurosporine displayed unique characteristics that implicate novel genetic circuitry in governing this morphogenetic transition. We discovered that filamentation triggered by staurosporine is mediated by the adenylyl cyclase Cyr1 and protein kinase PKA but is independent of the upstream regulators Ras1 and Pkc1 and largely independent of the downstream effectors Nrg1 and Efg1. Further, we found that septin ring and septum formation were defective in filaments formed in response to staurosporine, implicating perturbation of cytokinesis and suggesting cell cycle kinases as the relevant targets of staurosporine. Taken together, the findings in this work establish staurosporine as a tool compound to define the architecture of genetic circuitry central to morphogenetic regulation and illuminate novel functional relationships within a core pathway governing a key virulence trait.

## RESULTS

### Staurosporine induces morphological changes distinct from other filament-inducing cues.

**FIG 1:**
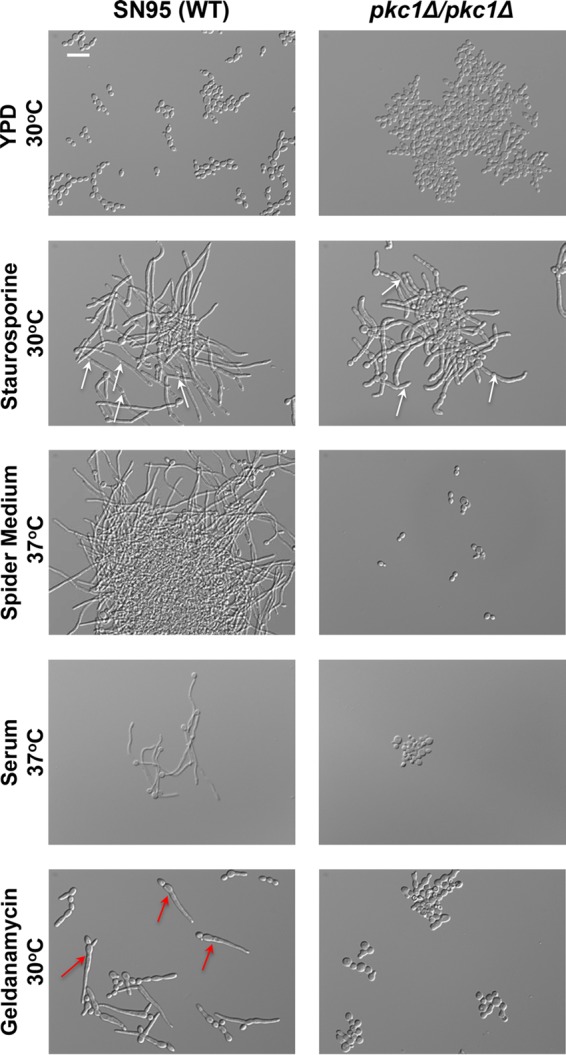
Staurosporine induces filamentation independent of Pkc1. SN95 wild-type (WT) cells were subcultured to log phase in YPD at 30°C, YPD plus 0.5 μg/ml staurosporine at 30°C, YPD plus 10 μM geldanamycin at 30°C, Spider medium at 37°C, or 10% serum at 37°C. Cells were imaged by DIC microscopy. The scale bar indicates 20 μm. White arrows highlight representative filaments with obvious constrictions along the filament. Red arrows highlight representative filaments with a widening at the bud neck that narrows toward the tip.

Staurosporine is a promiscuous protein kinase inhibitor commonly used to inhibit Pkc1. Recently we demonstrated that Pkc1 has a central role in governing the *C. albicans* yeast-to-filament transition (22). Thus, we sought to evaluate whether treatment of a wild-type strain of *C. albicans* with staurosporine would similarly block filamentous growth. Surprisingly, we discovered that staurosporine has the opposite impact and induced filamentous growth of a wild-type strain of *C. albicans* in rich medium at 30°C (Fig. 1). This is distinct from most conditions that induce filamentation, including carbon-limiting Spider medium or serum, which requires a concurrent increase in temperature to 37°C (see Fig. S1 in the supplemental material). A closer examination of the filaments induced by staurosporine revealed obvious constrictions along staurosporine-induced filaments, in contrast to true hyphae that lack constrictions as observed in Spider medium or in the presence of serum (Fig. 1). Staurosporine also induced filaments with distinct features from those induced by other cues that share the capacity to induce filamentation at 30°C, such as inhibition of the molecular chaperone Hsp90 with geldanamycin (14). Filaments formed upon treatment with geldanamycin display a widening at the bud neck that narrows toward the tip, but this progressive constriction was not observed in filaments induced by staurosporine (Fig. 1). Thus, staurosporine induces filaments with characteristics that implicate distinct cellular circuitry from that engaged by other filament-inducing cues.

10.1128/mSphere.00056-17.1 FIG S1  Filament-inducing cues such as Spider medium and serum require a concomitant increase in temperature to 37°C. A wild-type strain was subcultured in YPD plus 10% serum or Spider medium at 30°C or 37°C. The scale bar represents 20 μm. Download FIG S1, TIF file, 1.3 MB. Copyright © 2017 Xie et al. 2017 Xie et al. This content is distributed under the terms of the Creative Commons Attribution 4.0 International license.

Next, we tested whether homozygous deletion of* PKC1* blocks *C. albicans* filamentation in response to staurosporine, as it does in response to all other cues that we tested previously, including RPMI medium at 37°C, Spider medium at 37°C, 10% serum at 37°C, and geldanamycin at 30°C (22). Surprisingly, deletion of *PKC1* did not block filamentation in response to staurosporine (Fig. 1). Morphology of the filaments was similar to that observed for the wild type, with obvious constrictions along the staurosporine-induced filaments. To our knowledge, this is the first cue that is capable of inducing filamentous growth in a strain lacking* PKC1*. Collectively, this suggests that staurosporine promotes filamentation through distinct circuitry that is independent of Pkc1.

### Filamentation in response to staurosporine requires Cyr1 and PKA, but does not require other components of the cAMP signaling pathway.

**FIG 2:**
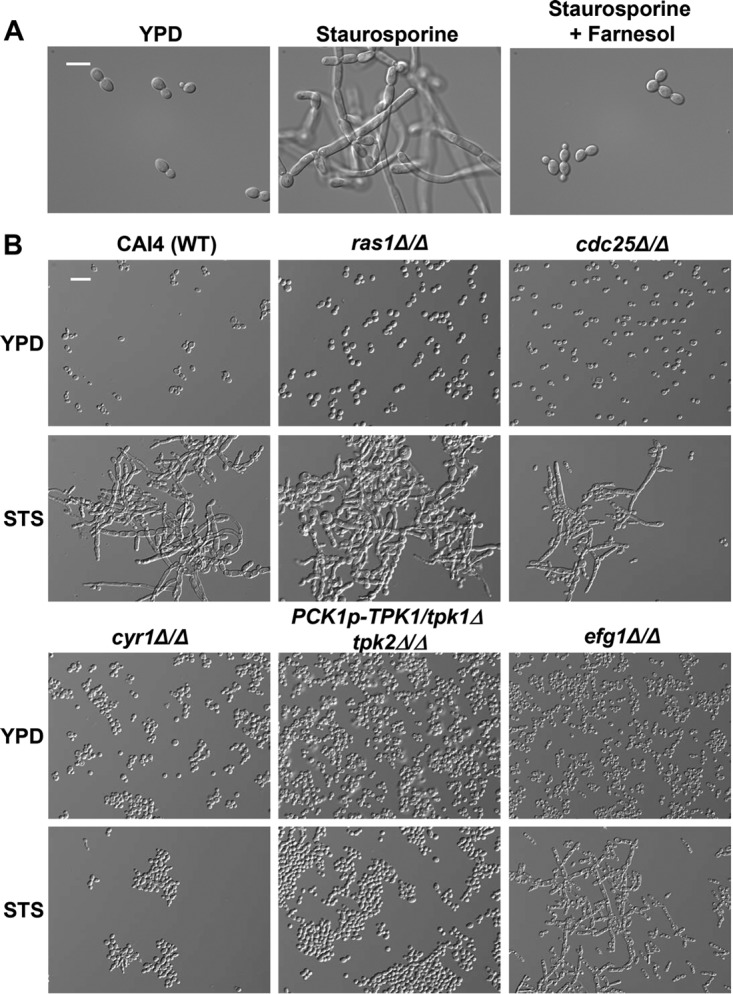
Staurosporine-induced filamentation requires Cyr1 and PKA. (A) Farnesol inhibits filamentation induced by staurosporine. SN95 wild-type cells were grown to log phase at 30°C in YPD or YPD plus 0.5 μg/ml staurosporine in the absence or presence of 200 μM farnesol. Cells were imaged by DIC microscopy. The scale bar indicates 10 μm. (B) Cyr1 and PKA are the only components of the cAMP signaling pathway tested that are required for filamentation induced by staurosporine. A CAI4 wild-type strain or mutants lacking components of the cAMP signaling pathway were subcultured to stationary phase in YPD at 30°C in the presence or absence of 0.5 μg/ml staurosporine (STS). Cells were imaged by DIC microscopy. The scale bar indicates 20 μm.

Given that Pkc1 was dispensable for staurosporine-induced filamentation, we tested the impact of perturbation of other morphogenetic regulators. We focused on the cAMP signaling cascade, which regulates the *C. albicans* yeast-to-filament transition in response to diverse cues, including serum and cell cycle arrest (13, 21, 31). To determine whether the cAMP signaling pathway is also required for filamentation induced by staurosporine, we tested the impact of farnesol, a quorum-sensing molecule that represses the yeast-to-filament transition through inhibition of the adenylyl cyclase Cyr1 (32). We found that farnesol blocked the induction of filamentation in response to staurosporine (Fig. 2A), suggesting that Cyr1 activity is important for staurosporine-induced filamentation. Next, we tested *C. albicans* mutants lacking components of the Ras1-Cyr1-PKA signaling pathway for their ability to filament in response to staurosporine; we tested mutants lacking *RAS1*, *CDC25*, *CYR1*, and *EFG1*, as well as a mutant with the catalytic subunits of PKA (*TPK1* and *TPK2*) depleted. We showed that both the wild-type CAI4 parental strain (Fig. 2B) as well as a uridine prototrophic wild-type control (see Fig. S2 in the supplemental material) filament robustly in response to staurosporine. We found that only Cyr1 and PKA were required for filamentation induced by staurosporine (Fig. 2B). Mutants lacking *RAS1* or *CDC25* were able to filament, suggesting that staurosporine can activate cAMP signaling independent of known regulators upstream of Cyr1 and PKA. Further, the mutant lacking *EFG1*, the key transcriptional regulator downstream of PKA required for morphogenesis in response to many cues, including serum, partially retained the ability to filament in response to staurosporine (Fig. 2B), suggesting that staurosporine signals through additional downstream targets of PKA. That Pkc1, Ras1, Cdc25, and Efg1 are largely dispensable, distinguishes morphogenesis in response to staurosporine from morphogenesis in response to other cues, highlighting additional unappreciated morphogenetic circuitry.

10.1128/mSphere.00056-17.2 FIG S2  Staurosporine induces robust filamentation in the wild-type strain CAF2-1. CAF2-1 was subcultured in YPD or YPD plus 0.5 μg/ml staurosporine at 30°C. The scale bar represents 20 μm. Download FIG S2, TIF file, 0.2 MB. Copyright © 2017 Xie et al. 2017 Xie et al. This content is distributed under the terms of the Creative Commons Attribution 4.0 International license.

### Filamentation in response to staurosporine is not accompanied by repression of yeast-specific genes or by degradation of the transcriptional repressor Nrg1.

**FIG 3:**
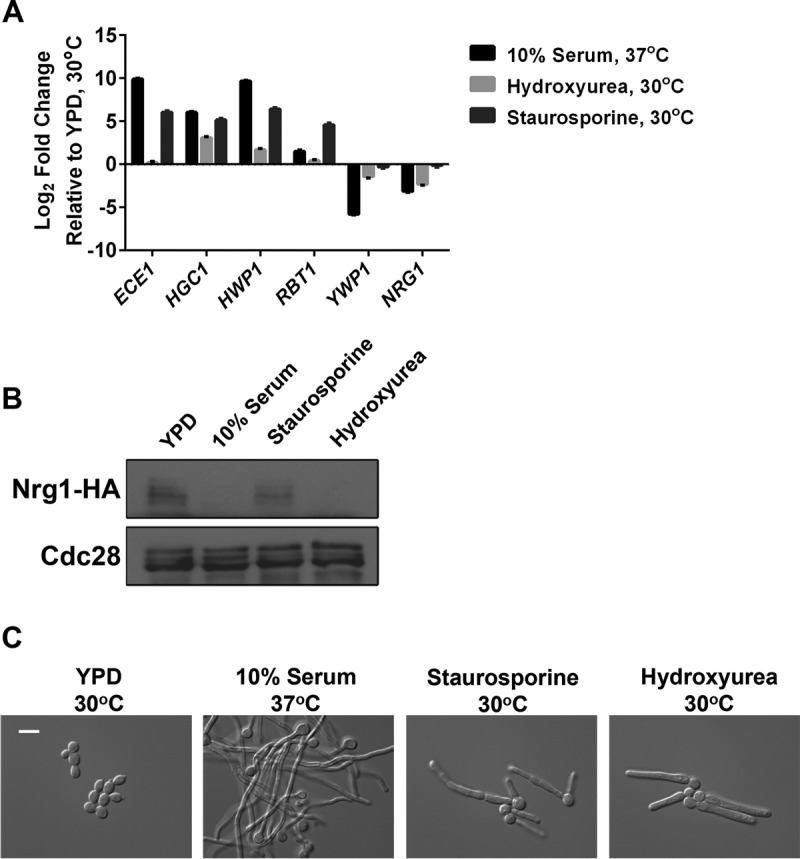
Staurosporine induces a distinct gene expression program from other filament-inducing cues and promotes filamentation independent of Nrg1 degradation. (A) SN95 wild-type cells were subcultured to log phase in YPD at 30°C, YPD plus 10% serum at 37°C, YPD plus 200 mM hydroxyurea at 30°C, or YPD plus 0.5 μg/ml staurosporine at 30°C. cDNA was prepared from total RNA for qRT-PCR. The transcript levels of filament-specific transcripts (*ECE1*, *HGC1*, *HWP1*, and *RBT1*) and yeast-specific transcripts (*YWP1* and *NRG1*) were monitored by qRT-PCR and normalized to *GPD1*. The fold change in gene expression under each condition relative to YPD at 30°C is plotted as the mean ± standard deviation from triplicate samples and is representative of two independent experiments. (B) Nrg1 protein persists in filaments induced by staurosporine. SN95 wild-type cells expressing native levels of HA-tagged Nrg1 were subcultured to log phase in YPD plus 10% serum at 37°C, YPD plus 200 mM hydroxyurea at 30°C, or YPD plus 0.5 μg/ml staurosporine at 30°C. Total protein was resolved by SDS-PAGE, and the blot was hybridized with anti-hemagglutinin to detect Nrg1 and anti-PSTAIRE to monitor Cdc28 as a loading control. (C) SN95 wild-type cells were grown to log phase under identical conditions as described for panel B. Cells were imaged by DIC microscopy. The scale bar indicates 10 μm.

Since activation of Cyr1 results in the induction of filament-specific genes and the repression of yeast-specific genes in response to diverse cues, we monitored the expression of a set of Cyr1-dependent transcripts in response to serum at 37°C, hydroxyurea at 30°C, and staurosporine at 30°C. We found that the patterns of expression of filament-specific transcripts *ECE1*, *HGC1*, *HWP1*, and *RBT1* were similar in filaments induced by serum or staurosporine but distinct in filaments induced by hydroxyurea (Fig. 3A). Strikingly, the yeast-specific transcripts *YWP1* and *NRG1* were not downregulated in response to staurosporine, unlike what was observed in the presence of serum (Fig. 3A). Thus, staurosporine activates a distinct transcriptional program relative to serum and hydroxyurea.

Next, we assessed levels of the transcriptional repressor Nrg1, for which degradation is thought to be required for initiation of filamentous growth (33). We monitored levels of hemagglutinin (HA)-tagged Nrg1 via Western blot analysis of filaments induced by serum, hydroxyurea, or staurosporine. Consistent with previous observations, Nrg1 was degraded upon growth for 4 h in serum or hydroxyurea (Fig. 3B); in contrast, Nrg1 levels persisted following treatment with staurosporine. This time point was sufficient to induce robust filamentous growth in the presence of each inducing cue (Fig. 3C). Similar results were observed at an earlier time point of 65 min posttreatment (see Fig. S3A in the supplemental material), during germ tube formation (Fig. S3B). Since Nrg1 is degraded in response to hydroxyurea-induced filamentation (Fig. 3B), the lack of Nrg1 degradation in response to staurosporine is not merely due to growth at 30°C. Our results suggest that staurosporine induces filamentation via distinct transcriptional regulatory control.

10.1128/mSphere.00056-17.3 FIG S3  Staurosporine induces filamentation independent of Nrg1 degradation. (A) Staurosporine-induced filamentation is not accompanied by Nrg1 protein degradation. SN95 wild-type cells expressing native levels of HA-tagged Nrg1 were grown in YPD plus 10% serum at 37°C, YPD plus 200 mM hydroxyurea (HU) at 30°C, or YPD plus 0.5 μg/ml staurosporine at 30°C for 5 or 65 min. Total proteins were resolved by SDS-PAGE gel, and the blot was hybridized with anti-HA to detect Nrg1 and anti-tubulin to monitor tubulin as a loading control. (B) Staurosporine induces germ tube formation at different kinetics from serum and hydroxyurea. Cells were imaged by DIC microscopy. The scale bar indicates 10 μm. Download FIG S3, TIF file, 1.3 MB. Copyright © 2017 Xie et al. 2017 Xie et al. This content is distributed under the terms of the Creative Commons Attribution 4.0 International license.

### Core mediators of *C. albicans* morphogenesis play limited roles in staurosporine-induced filamentation.

**FIG 4:**
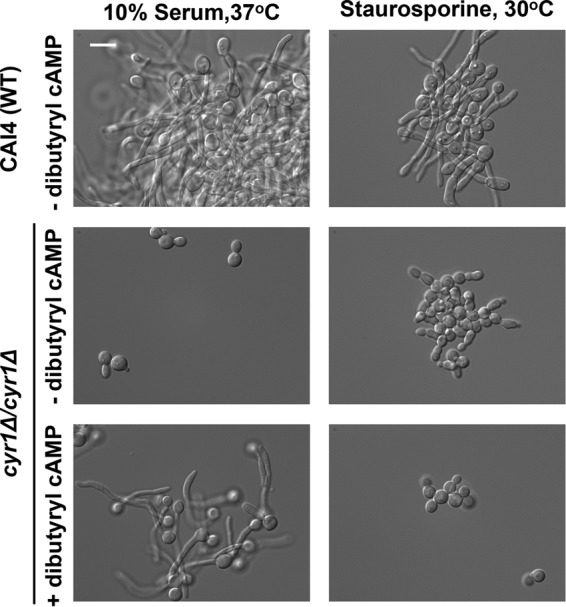
Dibutyryl cAMP does not rescue staurosporine-induced filamentation of a *cyr1*Δ/*cyr1*Δ mutant. A CAI4 wild-type strain and a *cyr1*Δ/*cyr1*Δ mutant were grown to log phase at 37°C in YPD plus 10% serum or at 30°C in YPD plus 0.5 μg/ml staurosporine in the absence or presence of 100 mM dibutyryl cAMP, as indicated. Cells were imaged by DIC microscopy. The scale bar indicates 10 μm.

Given our findings that filamentation in response to staurosporine requires Cyr1 and PKA but involves distinct transcriptional control, we tested whether signaling by the second messenger cAMP is important for staurosporine-induced filamentation. Production of cAMP by Cyr1 activates the catalytic subunits of PKA, Tpk1 and Tpk2, which in turn activate a transcriptional program that governs morphogenesis (12). In response to serum, the addition of dibutyryl cAMP, a nonhydrolyzable cAMP analogue, is sufficient to rescue filamentation in mutants unable to produce cAMP (34, 35). Strikingly, dibutyryl cAMP does not rescue filamentation of a *cyr1*Δ/*cyr1*Δ mutant in response to staurosporine, in stark contrast to the rescue observed in response to serum (Fig. 4). This suggests that cAMP signaling is not sufficient to support staurosporine-induced filamentous growth, or that the levels of cAMP required to induce the yeast-to-filament transition in response to staurosporine are higher than those required for other inducing cues. Both of these models implicate additional unidentified genetic factors in the establishment of polarized growth in response to staurosporine.

To identify additional genes required for staurosporine-induced filamentous growth, we performed a forward genetic screen of 1,248 *C. albicans* homozygous transposon insertion mutants that are enriched for protein kinases to identify strains that are unable to filament in response to this cue (36). Strikingly, we did not identify any mutants that were blocked in filamentation in response to staurosporine, suggesting either that this mutant library does not include the key regulator of staurosporine-induced filamentation or that staurosporine induces filamentous growth via multiple targets in *C. albicans*. To confirm that this library harbors mutants with disruption in genes involved in filamentous growth, we repeated the genetic screen to identify strains unable to filament in response to serum at 37°C. From this screen, two mutants (*upc2*Δ/*upc2*Δ and *rim13*Δ/*rim13*Δ) were completely blocked (filamentation score of 0) and nine mutants (*nup84*Δ/*nup84Δ*, *tpk2*Δ/*tpk2*Δ, *rfg1*Δ/*rfg1*Δ, *cka2*Δ/*cka2*Δ, *sok1*Δ/*sok1*Δ, *ire1*Δ/*ire1*Δ, *bck1*Δ/*bck1*Δ, *kin2*Δ/*kin2*Δ, and *ctm1*Δ/*ctm1*Δ) were partially blocked (filamentation score of 1) in filamentation in response to serum (see Table S1 in the supplemental material). These results demonstrate that this mutant library does harbor strains defective in filamentous growth, reinforcing that the circuitry governing morphogenesis in response to staurosporine is distinct from that implicated in response to serum.

10.1128/mSphere.00056-17.5 TABLE S1  Positive regulators of filamentation from the image-based arrayed morphology screen. Download TABLE S1, XLSX file, 0.1 MB. Copyright © 2017 Xie et al. 2017 Xie et al. This content is distributed under the terms of the Creative Commons Attribution 4.0 International license.

**FIG 5:**
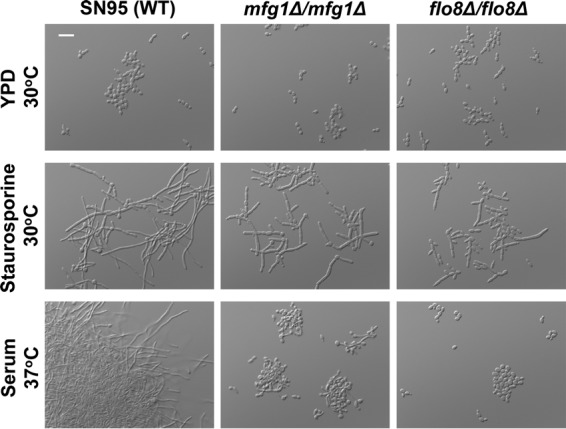
Mfg1 and Flo8 are not required for polarized growth in response to staurosporine. Strains were grown to log phase at 30°C in YPD, 30°C in YPD plus 0.5 μg/ml staurosporine, or 37°C in YPD plus 10% serum. Cells were imaged by DIC microscopy. The scale bar indicates 20 μm.

Next, we took a targeted approach and examined transcription factors with known roles in filamentation. Recently, a screen with a genome-wide set of targeted deletion alleles in *S. cerevisiae* identified a novel *m*orphogenetic regulator of *f*ilamentous *g*rowth gene, *MFG1*, with conserved roles in filamentation in *C. albicans* (37). Mfg1 binds to the transcription factors Flo8 and Mss11 to form a complex that is required for filamentation in response to serum (37). We confirmed the requirement of *MFG1* and *FLO8* for polarized growth in response to canonical inducing cues, as *mfg1*Δ/*mfg1*Δ and *flo8*Δ/*flo8*Δ mutants grew largely as yeast in the presence of serum (Fig. 5). In response to staurosporine, we found that the both the *mfg1*Δ/*mfg1*Δ and *flo8*Δ/*flo8*Δ mutants were able to initiate a polarized growth program, with the *flo8*Δ/*flo8*Δ mutant forming some chains of elongated yeast cells (Fig. 5). Together, this highlights the distinct genetic circuitry through which staurosporine induces morphogenesis in *C. albicans*.

### Staurosporine-induced filaments are defective in septin localization.

**FIG 6:**
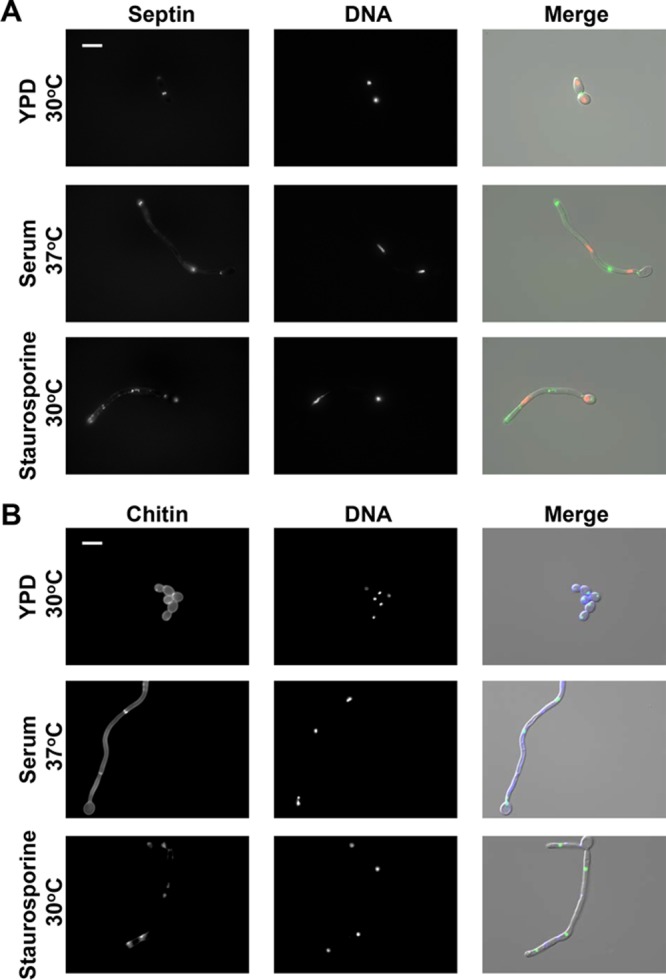
Septin ring formation and chitin-containing septum formation are aberrant in filaments formed in response to staurosporine. (A) Strains were subcultured to log phase in YPD at 30°C, YPD plus 0.5 μg/ml staurosporine at 30°C, or YPD plus 10% serum at 37°C. Shown are representative fluorescence microscopy images of SN95 wild-type cells expressing native levels of GFP-tagged Cdc10 to visualize septin (left panels) and RFP-tagged Hhf1 to visualize DNA (middle panels). The scale bar represents 10 μm. The fluorescence microscopy images were merged with DIC images (right panels). (B) Strains were subcultured to log phase in YPD at 30°C in the presence or absence of 0.5 μg/ml staurosporine. Shown are representative fluorescence microscopy images of SN95 wild-type cells expressing native levels of GFP-tagged Nop1 to visualize nuclei (middle panels) and stained with calcofluor white to visualize chitin (left panels). The scale bar represents 10 μm. The fluorescence microscopy images were merged with DIC images (right panels).

Given the distinct regulatory control of filamentation in response to staurosporine, we monitored the localization of septin. When hyphae are induced from yeast cells, a basal septin band appears transiently at the mother-germ tube junction (38, 39). Septin ring formation also occurs as the hyphal tip passes the site where septation will later occur (38, 39). Mitosis takes place across the plane of the septin ring, where nuclei eventually divide and localize to opposite poles of the septation site. When this process is complete, the septin ring separates into two rings and a primary septum composed of chitin is formed between them (39). To monitor septin localization in filaments formed in response to staurosporine, we generated a strain in which septin formation was marked by green fluorescent protein (GFP)-tagged Cdc10, and the nucleus was marked by red fluorescent protein (RFP)-tagged histone H4 (encoded by *HHF1*). Consistent with what has been previously reported, cells growing as yeast exhibited septin-GFP localization as a tight ring in between the mother-daughter bud (Fig. 6A). Nuclei were observed in both the mother and daughter cell, separated by the septum (Fig. 6A). Upon filamentation in response to serum, cell elongation and septum formation progressed successfully, as marked by the deposition of two septin rings and the separation of nuclei into individual compartments (Fig. 6A). However, in response to staurosporine, Cdc10-GFP puncta were misformed in areas with constrictions and were frequently observed throughout the filament, as opposed to remaining localized at the septation site (Fig. 6A). Nuclear division was also abnormal, with diffuse nuclear staining along the filament and some compartments missing nuclei altogether. Finally, chitin was either absent or was deposited in regions lacking constriction, as shown by the diffuse calcofluor white staining in staurosporine-induced filaments (Fig. 6B). The lack of septum formation suggests defects in cytokinesis (40), consistent with the observation that genetic and chemical perturbation of cell cycle progression often results in polarized growth (21). Thus, staurosporine may induce filamentation by inhibiting cell cycle kinases involved in cell division.

## DISCUSSION

The capacity to undergo morphological transitions is fundamental to sexual reproduction, nutrient acquisition, and virulence of diverse fungal species. This is exquisitely clear with the fungal pathogen *C. albicans*, for which the ability to transition between yeast and filamentous growth is a key virulence trait. We have uncovered a previously unappreciated effect of the promiscuous protein kinase inhibitor staurosporine on *C. albicans* morphogenesis. We discovered that staurosporine promotes filamentation independent of a concomitant increase in temperature to 37°C, as is required for the majority of inducing cues (Fig. 1). Further, to our knowledge, staurosporine is the only stimulus capable of inducing filamentation in the absence of the master morphogenetic regulator Pkc1 (Fig. 1). Similar to classical filament-inducing cues such as serum, staurosporine-induced filamentation requires the adenylyl cyclase Cyr1 and the cAMP-dependent protein kinase PKA; however, it does not require the upstream regulators of Cyr1, such as Pkc1 or Ras1 (Fig. 2). Further, the robust filamentation induced by staurosporine does not require downstream effectors of Cyr1 and PKA, such as *EFG1*, nor is it accompanied by the repression of Cyr1-dependent yeast-specific genes (Fig. 2B and 3A). We also demonstrate that staurosporine induces filamentation independent of degradation of the transcriptional repressor Nrg1 (Fig. 3B). Given that staurosporine causes defects in cell cycle progression and mislocalization of septin rings (Fig. 6), it is possible that staurosporine induces filamentation via repression of cell cycle kinases. The impact on morphogenesis could be mediated via inhibition of one of multiple cell cycle kinases that repress filamentation, including Cdc28, Cdc5, Cdc6, Cdc7, Ipl1, Mps1, Gin4, Hsl1, and Cak1 (20, 36, 41, 42). Notably, filamentation induced by inhibition of the kinase Cak1 occurs independently of Efg1, similar to what was observed in response to staurosporine (42). Our work exposes complexity in the genetic circuitry governing fungal morphogenesis and establishes staurosporine as a chemical probe to define circuitry controlling a key virulence trait.

Filaments induced by staurosporine exhibit a gene expression signature that diverges from the canonical filament-specific program and is likely a consequence of the distinct circuitry that mediates this cellular response. Staurosporine induces the expression of filament-specific transcripts, such as *HWP1* and *ECE1*, but does not repress yeast-specific transcripts, such as *YWP1* and *NRG1* (Fig. 3A). *YWP1* and *NRG1* are yeast-specific genes whose expression during yeast growth and repression during filamentation are both contingent on Efg1 (16, 43). The induction of filamentation in response to all cues explored to date is accompanied not only by transcriptional changes but also by degradation of Nrg1 (33). Strikingly, Nrg1 levels persist in filaments induced by staurosporine (Fig. 3). This is consistent with our finding that Efg1 is largely dispensable for staurosporine-induced filamentation and suggests that staurosporine initiates morphogenesis without Efg1 activation (Fig. 2). Polarized growth in response to staurosporine also occurs independent of the morphogenetic regulators Mfg1 and Flo8 (Fig. 5), implicating either a different transcription factor or redundancy at this level of transcriptional control. This divergence in genetic requirements for filamentation induced by different cues is further highlighted by regulators upstream of Cyr1. Filamentation induced by most cues requires not only Cyr1 and the cAMP-dependent kinase PKA, but also the upstream regulators Ras1 and Pkc1; in contrast, Ras1 and Pkc1 are dispensable for filamentation in response to staurosporine (Fig. 1 and 2), suggesting that staurosporine can activate the adenylyl cyclase independent of known activators, either due to redundancy or due to distinct sensors. This divergence in mechanisms of Cyr1 activation is further reinforced by the finding that dibutyryl cAMP rescues filamentation of a mutant lacking Cyr1 in response to serum, but not staurosporine (Fig. 4). This suggests either that elevated cAMP levels are required to restore filamentation in response to staurosporine compared to serum or that Cyr1 participates in an unappreciated cAMP-independent pathway important for morphogenesis. Nonetheless, this highlights complexity in the architecture of core cellular circuitry that controls responses to environmental cues.

The profound impact of staurosporine on cellular morphology extends beyond the fungal kingdom. Staurosporine induces cellular elongation in cultured mammalian cells (44), suggesting that the relevant target of staurosporine is conserved from yeast to humans. Notably, in mammalian cells, staurosporine induces actin reorganization independently of protein kinase C (45). Given that polymerization of actin is crucial for *C. albicans* filamentation (46, 47), the findings in mammalian cells suggest that staurosporine could promote fungal morphogenesis by modifying the actin cytoskeleton. Intriguingly, the cytotoxic effect of the actin polymerization inhibitor latrunculin B against *S. cerevisiae* can be suppressed by staurosporine, suggesting that the two compounds have opposing effects on actin dynamics (48). Further, studies in *S. cerevisiae* suggest that septin ring assembly can be impeded by actin perturbation. This resonates with our findings that staurosporine impairs septin ring formation (Fig. 6) and suggests that staurosporine could promote polarized growth by nucleating reorganization of the actin cytoskeleton.

There is emerging interest to utilize molecules targeting key hubs of cellular stress response in combination with current antifungals in order to prolong the life span of existing therapeutics for the treatment of serious fungal infections. This interest is based on the observation that multiple small molecules that potentiate the activity of antifungal drugs also modulate fungal morphology. For example, pharmacological inhibition of Hsp90 with natural products such as geldanamycin or radicicol abrogates resistance to azoles and echinocandins, and induces filamentous growth at 30°C (14). Similarly, the metal chelator DTPA (diethylenetriaminepentaacetic acid) potentiates echinocandin activity both *in vitro* and *in vivo* via depletion of magnesium and induces polarized growth at 30°C via depletion of zinc (49). The natural product beauvericin provides another example of a molecule that potentiates azole activity via inhibition of TOR (*t*arget *o*f *r*apamycin) signaling and multidrug efflux, but it blocks morphogenesis in response to diverse cues (6). Staurosporine expands the repertoire of chemical scaffolds that influence both drug resistance and virulence traits; it enhances the efficacy of azoles and echinocandins at least in part via inhibition of Pkc1 (3) and induces fungal morphogenesis at 30°C via a mechanism that is independent of Pkc1 and involves Cyr1 and PKA (Fig. 1 and 2). The next frontier will be to further define the specific targets engaged by these compounds, in order to enable structure-guided design of improved specificity and fungal selectivity in the development of much needed novel therapeutics for treatment of life-threatening fungal infections.

## MATERIALS AND METHODS

### Strains and reagents.

All *C. albicans* strains were archived in 25% glycerol and stored at −80°C. Overnight cultures were grown in YPD (1% yeast extract, 2% Bacto peptone, 2% dextrose) at 30°C. Two percent agar was added for solid media. Strains were constructed according to standard protocols. Strain construction is described in Text S1 in the supplemental material, and the strains used in this study are listed in Table S2. Staurosporine (AG Scientific, Inc., catalog no. S-1016) was formulated at 1 mg/ml in dimethyl sulfoxide (DMSO) and used at a final concentration of 0.5 μg/ml. Geldanamycin (Cedarlane catalog no. ant-gl-5) was formulated at 5 mM in DMSO and used at a final concentration of 10 μM. Hydroxyurea (BioShop Canada, Inc.) was formulated at 2 M in H_2_O, filter sterilized, and used at a final concentration of 200 mM. Dibutyryl cAMP (Sigma-Aldrich catalog no. D0627) was formulated at 100 mg/ml and used at a final concentration of 10 mg/ml. Fluorescent brightener 28, also known as calcofluor white (Sigma-Aldrich catalog no. F3543), was formulated at 10 mg/ml and used at a final concentration of 1 μg/ml.

10.1128/mSphere.00056-17.4 TEXT S1  Strain and plasmid construction. Download TEXT S1, DOCX file, 0.1 MB. Copyright © 2017 Xie et al. 2017 Xie et al. This content is distributed under the terms of the Creative Commons Attribution 4.0 International license.

### Culture conditions.

To compare and contrast cellular morphologies in response to different filament-inducing cues by microscopy, cells were subcultured for 4 h in YPD at 30°C, YPD with 10% (vol/vol) heat-inactivated newborn calf serum (NBCS [Gibco catalog no. 26010-066]) at 37°C, or Spider medium (1% mannitol, 1% nutrient broth and 0.2% K_2_HPO_4_), or were subcultured for 6 h in YPD with 10 μM geldanamycin at 30°C, or YPD with 0.5 μg/ml staurosporine. To monitor gene expression in response to filament-inducing cues by quantitative reverse transcription-PCR (qRT-PCR), cells were subcultured in YPD at 30°C, YPD with 10% serum, YPD with 200 mM hydroxyurea, or YPD with 0.5 μg/ml staurosporine at an optical density at 600 nm (OD_600_) of 0.15 and grown for 4 h. To monitor Nrg1 protein levels by Western blotting, cells were subcultured in YPD at 30°C, YPD with 10% serum, YPD with 200 mM hydroxyurea, or YPD with 0.5 μg/ml staurosporine at an OD_600_ of 0.2 and grown for 5 min, 65 min, or 4 h, as indicated.

### Plasmid construction.

Plasmid construction is described in Text S1, the plasmids used in this study are listed in Table S3, and the primers used in this study are listed in Table S4 in the supplemental material.

10.1128/mSphere.00056-17.6 TABLE S2  *Candida albicans* strains used in this study. Download TABLE S2, DOCX file, 0.1 MB. Copyright © 2017 Xie et al. 2017 Xie et al. This content is distributed under the terms of the Creative Commons Attribution 4.0 International license.

10.1128/mSphere.00056-17.7 TABLE S3  Plasmids used in this study. Download TABLE S3, DOCX file, 0.1 MB. Copyright © 2017 Xie et al. 2017 Xie et al. This content is distributed under the terms of the Creative Commons Attribution 4.0 International license.

10.1128/mSphere.00056-17.7 TABLE S4  Oligonucleotides used in this study. Download TABLE S4, XLSX file, 0.1 MB. Copyright © 2017 Xie et al. 2017 Xie et al. This content is distributed under the terms of the Creative Commons Attribution 4.0 International license.

### qRT-PCR.

To prepare samples for RNA extraction, 10 ml of culture was harvested by centrifugation at 2095 g for 5 min at 4°C. The pellet was flash frozen and stored at −80°C overnight. RNA was isolated using the Qiagen RNeasy kit, and cDNA was generated using the AffinityScript cDNA synthesis kit (Stratagene). qRT-PCR was carried out using the Fast SYBR green master mix (Thermo Fisher Scientific) in 384-well plates with the following cycle conditions: 95°C for 10 min with a repeat at 95°C for 10 s and then 60°C for 30 s for 40 cycles. The melt curve was completed with the following cycle conditions: 95°C for 10 s and 65°C for 5 s with an increase of 0.5°C per cycle up to 95°C. All reactions were done in triplicate. Data were analyzed in the Bio-Rad CFX manager 3.1.

### Western blotting.

To prepare samples for protein extraction, 10 ml of culture was harvested by centrifugation at 2,095 × *g* for 5 min at 4°C. The pellet was resuspended in 50 μl of sample buffer (0.167 vol of 6× sample buffer containing 0.35 M Tris-HCl, 10% [wt/vol] SDS, 36% glycerol, 5% β-mercaptoethanol, and 0.012% bromophenol blue). The samples were boiled for 5 min at 95°C. The cell debris was spun down, and the supernatant was resolved on a 10% SDS-PAGE gel to monitor changes in Nrg1 levels. Separated proteins were electrotransferred to polyvinylidene difluoride (PVDF) membrane (Bio-Rad Laboratories, Inc.) and blocked with 5% skim milk in phosphate-buffered saline (PBS) with 0.2% Tween 20 at room temperature for 1 h. Blots were hybridized with primary antibody against the HA epitope (1:5,000 dilution; Roche Diagnostics), α-tubulin (1:1,000; AbD Serotec no. MCA78G), or α-PSTAIRE (1:1,000; Santa Cruz Biotechnology) overnight at 4°C. Blots were washed with PBS with 0.1% Tween 20 and subsequently hybridized with fluorescein isothiocyanate (FITC)-conjugated secondary antibody diluted 1:5,000 in the block solution for 45 min at room temperature. Signals were detected using an ECL enhanced chemiluminescence Western blotting kit as per the manufacturer’s instructions (Pierce).

### Microscopy.

To monitor *C. albicans* morphogenesis, images were captured using differential interference contrast (DIC) microscopy on a Zeiss Imager M1 upright microscope and AxioCam Mrm with AxioVision 4.7 software at 40× or ×100 magnification. For fluorescence microscopy, an X-cite series 120 light source with ET green fluorescent protein (GFP), 4′,6-diamidino-2-phenylindole (DAPI) hybrid, and ET HQ tetramethylrhodamine isothiocyanate (TRITC)/DsRED filter sets from Chroma Technology (Bellows Falls, VT) was used. Calcofluor white was viewed under the DAPI hybrid filter, RFP-tagged Hhf1 was viewed under the Texas Red filter, and GFP-tagged Nop1 and GFP-tagged Cdc10 were viewed under the GFP filter. To monitor chitin localization, 1 ml of culture was centrifuged at 16,000 × *g* for 1 min. The supernatant was removed, and the cells were washed with PBS. Calcofluor white was added to a final concentration of 1 µg/ml in a final volume of 100 µl. The cells were incubated in the dark for 15 min and were gently vortexed every 3 to 4 min. Cells were washed with PBS, and 2 µl of the cells was deposited on a cover slide for viewing under the microscope.

### Arrayed morphology screen with staurosporine.

A *C. albicans* homozygous mutant library generated by transposon insertion (36) was screened by microscopy. Overnight cultures were set up by inoculating 200 μl of YPD with the mutant library in 96-well plates at 30°C. Approximately 0.5 μl of cells was then transferred into either YPD only or YPD with 0.5 μg/ml of staurosporine using a 96-well pinner. The plates were incubated at 30°C for 4 h under static conditions. Images of potential hits were captured on a Zeiss Axio Observer.Z1 (Carl Zeiss, Inc.) using ×10 and ×40 magnifications and validated in a similar experimental setup. The criterion for a hit was a reproducible block in filamentous growth. If independent mutants in the library existed for the same open reading frame, both had to exhibit a block in filamentation in response to staurosporine to be considered a hit.

### Arrayed morphology screen with serum.

Strains were inoculated into 96-well plates overnight. To induce filamentation, cells were pinned into 100 µl of fresh medium in either YPD or YPD plus 10% newborn calf serum, and the plates were incubated for 4 h at 30°C or 37°C, respectively. Images were then taken on an AxioObserver inverted microscope. Each image was then scored for degree of filamentation, where 0 is indicative of an entirely yeast population and 3 indicates strains with wild-type levels of filamentation in response to serum. If independent mutants in the library existed for the same open reading frame, both had to exhibit a block in filamentation in response to serum to be considered a hit.
