# Supplementary figures and images for: Staurosporine Induces Filamentation in the Human Fungal Pathogen Candida albicans via Signaling through Cyr1 and Protein Kinase A
Source: mSphere. 2017 Mar 1;2(2):e00056-17. doi: 10.1128/mSphere.00056-17 (PMC5332603; doi:10.1128/mSphere.00056-17)

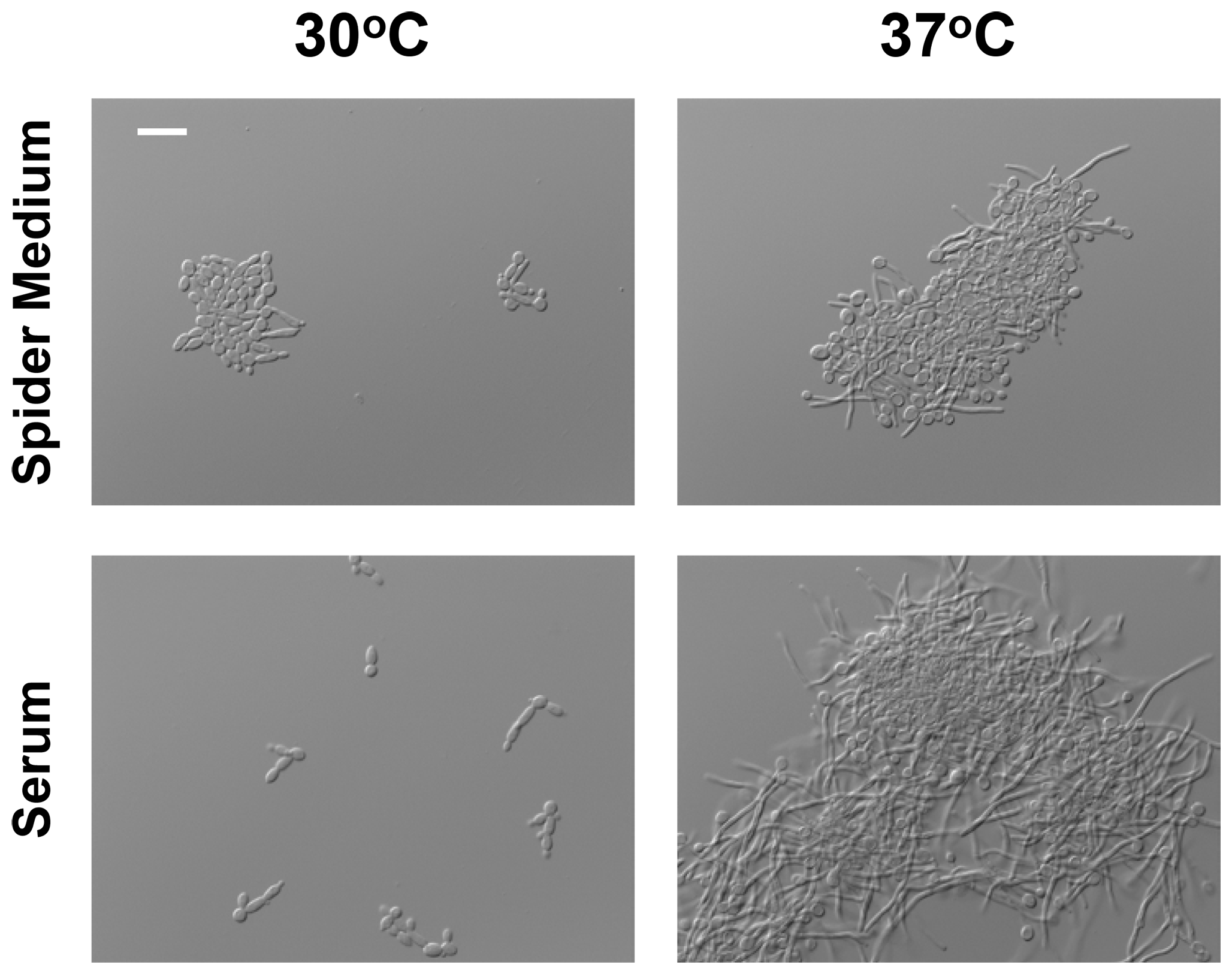

Supplement: FIG S1 [file sph002172243sf1.tif]

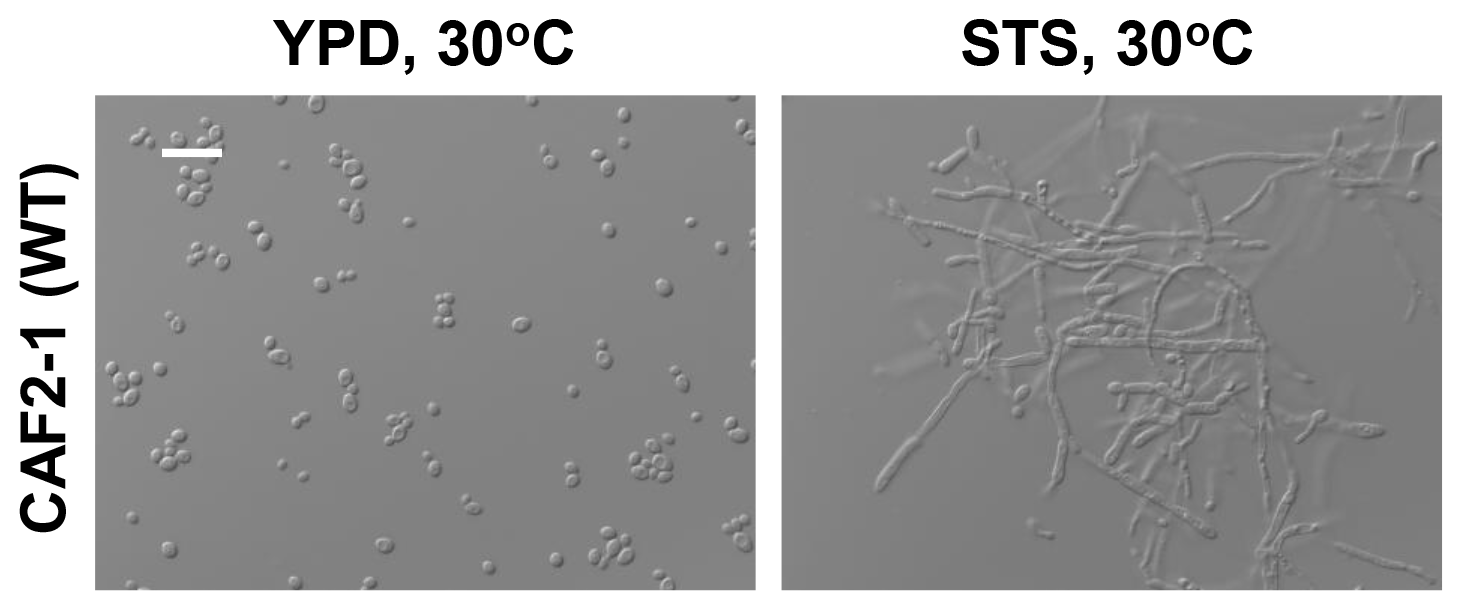

Supplement: FIG S2 [file sph002172243sf2.tif]

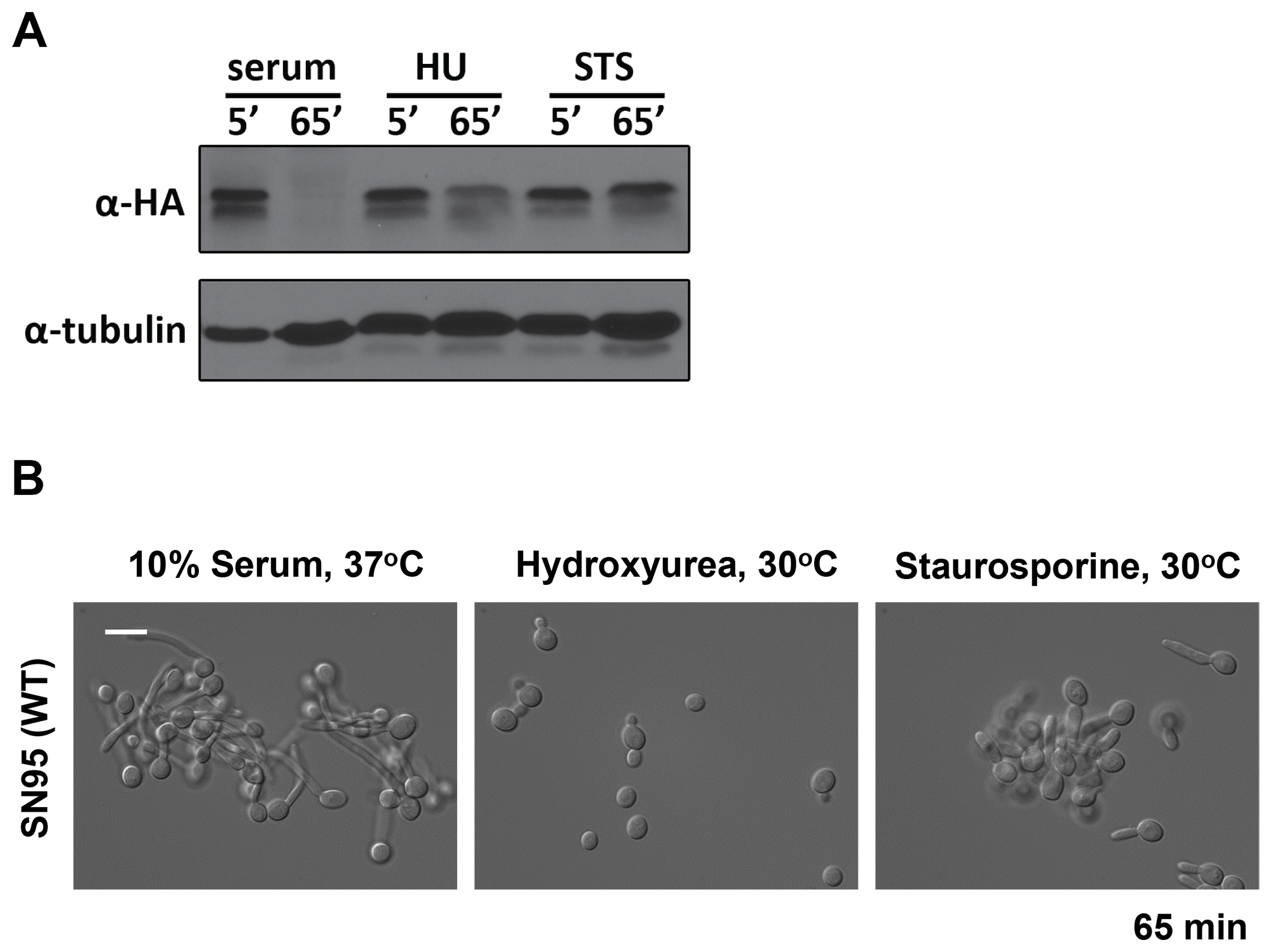

Supplement: FIG S3 [file sph002172243sf3.tif]
