# Supplementary material for: Staurosporine Induces Filamentation in the Human Fungal Pathogen Candida albicans via Signaling through Cyr1 and Protein Kinase A
Source: mSphere. 2017 Mar 1;2(2):e00056-17. doi: 10.1128/mSphere.00056-17 (PMC5332603; doi:10.1128/mSphere.00056-17)
Supplement: TEXT S1 [file sph002172243s4.docx]

**Supplemental Text**

**Strain Construction**

**CaLC2897**: To generate a *FLO8* heterozygous deletion mutant, the NAT flipper cassette (pLC49) (1) was PCR amplified using primers oLC2818 and oLC2819 (4366 bp) and transformed into CaLC239. NAT-resistant transformants were PCR tested for proper integration with oLC275 + oLC2820 (upstream 594 bp) and oLC274 + oLC2821 (downstream 793 bp). The *SAP2* promoter was induced to drive expression of FLP recombinase to excise the NAT flipper cassette. To generate a *FLO8* homozygous deletion mutant, the NAT flipper cassette (pLC49) (1) was PCR amplified using primers oLC2818 and oLC2819 (4366 bp) and transformed into the *FLO8* heterozygous deletion mutant. NAT-resistant transformants were PCR tested for proper integration with oLC275 + oLC2820 (upstream 594 bp) and oLC274 + oLC2821 (downstream 793 bp). To verify that the first allele was still deleted, primers oLC2820 + oLC2821 were used. Absence of the wild-type allele was verified using primers oLC2820 + oLC2846. The *SAP2* promoter was induced to drive expression of FLP recombinase to excise the NAT flipper cassette.

**CaLC4697:** To C-terminally tag Hhf1 with RFP, the RFP-NAT cassette was PCR amplified from pLC447 using primers oLC4752 and oLC4753 (3883 bp) and transformed into CaLC239. Correct integration downstream of *HHF1* was verified by amplifying across both junctions using primer pairs oLC4417 + oLC4754 (upstream 879 bp) and oLC274 + oLC4755 (downstream 239 bp).

**CaLC4711:** To C-terminally tag Cdc10 with GFP, the GFP-HIS cassette was PCR amplified from pLC383 (2) using primers oLC4770 and oLC4771 (>5 kb) and transformed into CaLC4697. Correct integration downstream of *CDC10* was verified by amplifying across both junctions using primer pairs oLC600 + oLC4772 (upstream 471 bp) and oLC1645 + oLC4773 (downstream <600 bp).

**CaLC4506:** To C-terminally tag Nop1 with GFP, the GFP-HIS cassette was PCR amplified from pLC383 (2) using primers oLC4415 and oLC4418 (>5 kb) and transformed into CaLC239. Correct integration at the C-terminus of NOP1 was verified by amplifying across both junctions using primer pairs oLC600 + oLC4400 (upstream 526 bp) and oLC4401 + oLC1645 (downstream ~1kb).

**Plasmid Construction**

**pLC447:** This is a construct for C-terminally tagging protein with RFP using nourseothricin (NAT) as the selective marker. Cherry/RFP was PCR amplified from pLC435 (3) with oLC841/oLC842 and digested with BsrGI. This was cloned into pLC389 (2) at BsrGI. Directionality of the insert was verified by PCR with oLC849 and oLC842. Clones were sequenced with oLC849 and oLC842.

**Supplemental References**

1. Morschhauser J, Michel S, Staib P. 1999. Sequential gene disruption in Candida albicans by FLP-mediated site-specific recombination. Mol Microbiol 32:547-56.

2. Gerami-Nejad M, Berman J, Gale CA. 2001. Cassettes for PCR-mediated construction of green, yellow, and cyan fluorescent protein fusions in *Candida albicans*. Yeast 18:859-64.

3. Keppler-Ross S, Noffz C, Dean N. 2008. A new purple fluorescent color marker for genetic studies in *Saccharomyces cerevisiae* and *Candida albicans*. Genetics 179:705-10.

4. Noble SM, Johnson AD. 2005. Strains and strategies for large-scale gene deletion studies of the diploid human fungal pathogen *Candida albicans*. Eukaryot Cell 4:298-309.

5. LaFayette SL, Collins C, Zaas AK, Schell WA, Betancourt-Quiroz M, Gunatilaka AA, Perfect JR, Cowen LE. 2010. PKC signaling regulates drug resistance of the fungal pathogen *Candida albicans* via circuitry comprised of Mkc1, calcineurin, and Hsp90. PLoS Pathog 6:e1001069.

6. Fonzi WA, Irwin MY. 1993. Isogenic strain construction and gene mapping in *Candida albicans*. Genetics 134:717-28.

7. Jain P, Akula I, Edlind T. 2003. Cyclic AMP signaling pathway modulates susceptibility of *Candida* species and *Saccharomyces cerevisiae* to antifungal azoles and other sterol biosynthesis inhibitors. Antimicrob Agents Chemother 47:3195-201.

8. Bockmuhl DP, Krishnamurthy S, Gerads M, Sonneborn A, Ernst JF. 2001. Distinct and redundant roles of the two protein kinase A isoforms Tpk1p and Tpk2p in morphogenesis and growth of *Candida albicans*. Mol Microbiol 42:1243-57.

9. Ryan O, Shapiro RS, Kurat CF, Mayhew D, Baryshnikova A, Chin B, Lin ZY, Cox MJ, Vizeacoumar F, Cheung D, Bahr S, Tsui K, Tebbji F, Sellam A, Istel F, Schwarzmuller T, Reynolds TB, Kuchler K, Gifford DK, Whiteway M, Giaever G, Nislow C, Costanzo M, Gingras AC, Mitra RD, Andrews B, Fink GR, Cowen LE, Boone C. 2012. Global gene deletion analysis exploring yeast filamentous growth. Science 337:1353-6.

10. Xie JL, Grahl N, Sless T, Leach MD, Kim SH, Hogan DA, Robbins N, Cowen LE. 2016. Signaling through Lrg1, Rho1 and Pkc1 governs *Candida albicans* morphogenesis in response to diversecues. PLoS Genet 12:e1006405.
