# Supplementary material for: Staurosporine Induces Filamentation in the Human Fungal Pathogen Candida albicans via Signaling through Cyr1 and Protein Kinase A
Source: mSphere. 2017 Mar 1;2(2):e00056-17. doi: 10.1128/mSphere.00056-17 (PMC5332603; doi:10.1128/mSphere.00056-17)
Supplement: TABLE S2 [file sph002172243st6.docx]

**Table S2. *Candida albicans* strains used in this study.**

| Strain Name | Genotype or phenotype | Source |
| --- | --- | --- |
| CaLC239 (SN95) | *arg4∆/arg4∆ his1∆/his1∆ URA3/ura3∆::imm434 IRO1/iro1::imm434* | (4) |
| CaLC206 | SN95 *CaTAR-FRT∷his1∆/his1∆* | (4) |
| CaLC948 | CaLC206 *PKC1/pkc1∷FRT* | (5) |
| CaLC1255 | CaLC206 *pkc1∷FRT/pkc1∷FRT* | (5) |
| CaLC75 (CAI4) | *ura3::imm434/ura3::imm434* | (6) |
| CaLC2742 (CAF2-1) | *ura3::imm434/URA3* | (6) |
| CaLC564 | CAI4 *ras1::hisG-URA3-hisG/ras1::hisG* | (7) |
| CaLC558 | CAI4 *cdc25::hisG-URA3-hisG/cdc25::hisG* | (7) |
| CaLC555 | CAI4 *cyr1::hisG-URA3-hisG/cyr1::hisG* | (7) |
| CaLC843 | CAI4 *URA3-PCK1p-TPK1/tpk1::hisG tpk2::hisG/tpk2::hisG* | (8) |
| CaLC563 | CAI4 *efg1::hisG-URA3-hisG/efg1::hisG* | (7) |
| CaLC1909 | SN95 *mfg1∆/mfg1∆* | (9) |
| CaLC2897 | SN95 *flo8∆/flo8∆* | This study |
| CaLC4697 | SN95 *HHF1-RFP-NAT/HHF1* | This study |
| CaLC4711 | SN95 *CDC10-GFP-HIS/CDC10 HHF1-RFP-NAT/HHF1* | This study |
| CaLC4132 | SN95 *NRG1-HA-HIS/NRG1* | (10) |
| CaLC4506 | SN95 *NOP1-GFP-HIS/NOP1* | This study |
