# Supplementary material for: Staurosporine Induces Filamentation in the Human Fungal Pathogen Candida albicans via Signaling through Cyr1 and Protein Kinase A
Source: mSphere. 2017 Mar 1;2(2):e00056-17. doi: 10.1128/mSphere.00056-17 (PMC5332603; doi:10.1128/mSphere.00056-17)
Supplement: TABLE S3 [file sph002172243st7.docx]

**Table S3. Plasmids used in this study.**

| Strain Name | Description | Source |
| --- | --- | --- |
| pLC49 | *FLP-CaNAT, ampR* | (1) |
| pLC447 | CaCherry-NAT, *ampR* | This study |
| pLC383 | GFP-HIS, *ampR* | (2) |
| pLC389 | GFP-NAT, *ampR* | (2) |
| pLC435 | Clp1-ADH1-Cherry, *URA3* | (3) |
